# 8-[(2-Hy­droxy­phen­yl)imino]-3,5a,9-trimethyl-3a,4,5,5a,8,9b-hexa­hydro­naphtho­[1,2-*b*]furan-2(3*H*)-one

**DOI:** 10.1107/S1600536812027146

**Published:** 2012-06-20

**Authors:** Sammer Yousuf, Syed M. Younas, Nida Ambreen, Khalid M. Khan, Ghulam A. Miana

**Affiliations:** a H.E.J. Research Institute of Chemistry, International Center for Chemical and Biological Sciences, University of Karachi, Karachi 75270, Pakistan; b Department of Chemistry, Allama Iqbal Open University, Islamabad; c Riphah Institute of Pharmaceutical Sciences, Riphah International University, 7th Avenue G-7/4, Islamabad, Pakistan

## Abstract

The title compound, C_21_H_23_NO_3_, is a phenyl­imine derivative of the well known anthelmintic agent α-santonin. The *trans*-fused cyclo­hexane and γ-lactone rings of the α-santonin ring system adopt chair and envelope conformations, respectively, whereas the hexa­diene ring is approximately planar [maximum deviation = 0.029 (4) Å] and forms a dihedral angle of 62.30 (11)° with the benzene ring. An intra­molecular O—H⋯N hydrogen bond is observed.

## Related literature

For the isolation and anthelmintic use of *α*-santonin, see: Miana & Al-Lohedan (1986 ▶). For the crystal structure and stereochemistry of *α*-santonin, see: White & Sim (1975 ▶); Coggon & Sim (1969 ▶). For the crystal structure of a related compound, see: Yousuf *et al.* (2012 ▶).
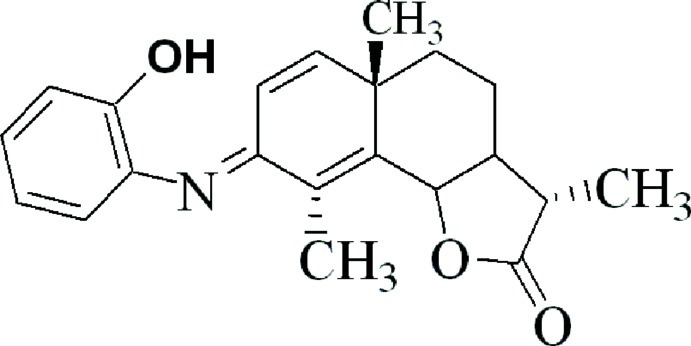


## Experimental

### 

#### Crystal data

C_21_H_23_NO_3_
*M* *_r_* = 337.40
Orthorhombic, 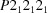

*a* = 8.6000 (9) Å
*b* = 10.7458 (11) Å
*c* = 19.729 (2) Å
*V* = 1823.2 (3) Å^3^
*Z* = 4
Mo *K*α radiation
μ = 0.08 mm^−1^
*T* = 273 K
0.54 × 0.14 × 0.04 mm

#### Data collection

Bruker SMART APEX CCD area-detector diffractometer
Absorption correction: multi-scan (*SADABS*; Bruker, 2000 ▶) *T*_min_ = 0.957, *T*_max_ = 0.997
10874 measured reflections
1955 independent reflections
1385 reflections with *I* > 2σ(*I*)
*R*_int_ = 0.055

#### Refinement

*R*[*F* ^2^ > 2σ(*F* ^2^)] = 0.041
*wR*(*F* ^2^) = 0.101
*S* = 1.04
1955 reflections
228 parameters
H-atom parameters constrained
Δρ_max_ = 0.14 e Å^−3^
Δρ_min_ = −0.13 e Å^−3^

### 

Data collection: *SMART* (Bruker, 2000 ▶); cell refinement: *SAINT* (Bruker, 2000 ▶); data reduction: *SAINT*; program(s) used to solve structure: *SHELXS97* (Sheldrick, 2008 ▶); program(s) used to refine structure: *SHELXL97* (Sheldrick, 2008 ▶); molecular graphics: *SHELXTL* (Sheldrick, 2008 ▶); software used to prepare material for publication: *SHELXTL*, *PARST* (Nardelli, 1995 ▶) and *PLATON* (Spek, 2009 ▶).

## Supplementary Material

Crystal structure: contains datablock(s) global, I. DOI: 10.1107/S1600536812027146/rz2770sup1.cif

Structure factors: contains datablock(s) I. DOI: 10.1107/S1600536812027146/rz2770Isup2.hkl

Supplementary material file. DOI: 10.1107/S1600536812027146/rz2770Isup3.cml

Additional supplementary materials:  crystallographic information; 3D view; checkCIF report

## Figures and Tables

**Table 1:** Hydrogen-bond geometry (Å, °)

*D*—H⋯*A*	*D*—H	H⋯*A*	*D*⋯*A*	*D*—H⋯*A*
O3—H3*A*⋯N1	0.82	2.28	2.747 (4)	116

**Fig. 1.**
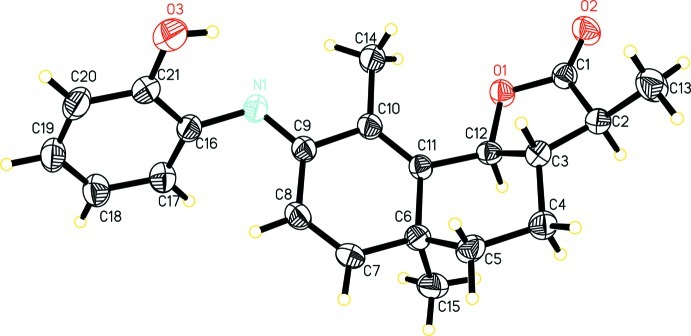
The molecular structure of the title compound with displacement ellipsoids drawn at 30% probability level.

**Fig. 2.**
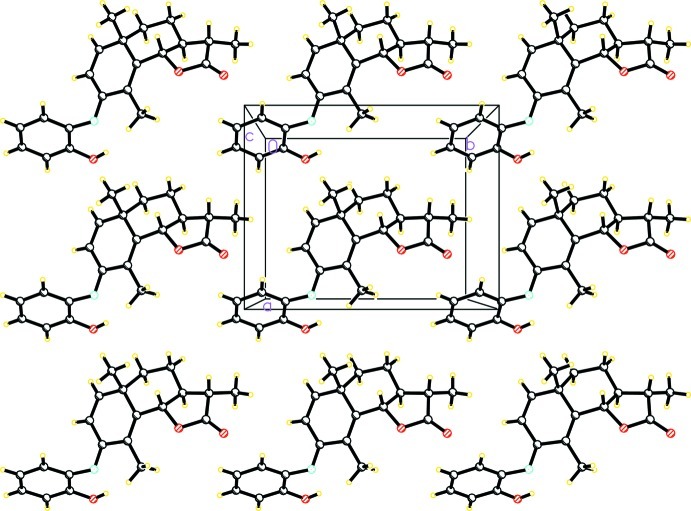
The crystal packing of the title compound viewed down the c axis.

## supplementary crystallographic information

### Comment

*α*-Santonin was isolated from *Artemisia santonica* (Miana & Al-Lohedan, 1986) and widely used in the past as an anthelmintic drug to expels parasitic worms (helminths) from the body, by either killing or stunning them. The title compound was prepared as a part of our ongoing reaserch to synthesize bioactive derivatives of *α*-santonin *via* biology oriented synthesis (BIOS). The title compound is an analogue of our previously reported compound 3,5a,9-trimethyl-3a,5,5a,9b-tetrahydronaphtho[1,2-*b*]furan-2,8(3*H*,4*H*)-dione-8-(*N*-phenylhydrazone), with the difference that the phenylhydrazine moiety is replaced by a 2-hydroxyphenylimine group (C16–C21) attached to the *α*-santonin ring system (O1–O2/C1–C15). The cyclohexadiene ring (C6–C11) is almost planar with a maximum deviation from the least square plane of 0.029 (3) Å for atom C7 and forms a dihedral angle of 62.30 (11)° with the phenyl ring. The cyclohexane ring (C3–C6/C11–C12) adopts a chair conformation [*Q* = 0.594 (4) Å, θ = 8.2 (4)° and φ = 304 (2)°] and is *trans* fused to the *γ*-lactone ring (O1/C1–C3/C12) which adopts an envelope conformation with atom C3 0.228 (3) Å out of the plane formed by the rest of the ring atoms. The two methyl substituents at atoms C6 and C2 exist in *axial* and *pseudo equatorial* orientations, respectively (Fig. 1). The bond dimensions are similar to those found in the structurally related compounds (Yousuf *et al.*, 2012; White & Sim, 1975; Coggon & Sim, 1969). An intramolecular O—H···N hydrogen bond is present (Table 1). In the crystal, molecules are arranged into layers parallel to the *ab* plane only by van der Waals forces (Fig. 2).

### Experimental

In a 100 ml round bottomed flask toluene (25 ml) and *α*-santonin (400 mg, 1.6 mmol) were taken, then 2-amino phenol (11.2 mmol) was added with continuous stirring. The reaction mixture was refluxed and monitored by TLC. After completion of reaction (24 h), the mixture was cooled and extracted with water. The organic layer was dried over Na_2_SO_4_, filtered and the solvent evaporated under vacuum in a rotary evaporator. The crude product was chromatographed on a silica gel column using *n*-hexane:ethyl acetate (7:3 *v*/*v*) as mobile phase to obtain yellow crystals of title compound in 85% yield.

### Refinement

H atoms were positioned geometrically with C—H = 0.93–0.97 Å, O—H = 0.82 Å, and constrained to ride on their parent atoms with *U*_iso_(H)= 1.2 *U*_eq_(C) or 1.5*U*_eq_(C, O) for methyl and hydroxy H atoms. A rotating group model was applied to the methyl groups. 1433 Friedel pairs were merged.

### Crystal data

**Table:**

C_21_H_23_NO_3_	*F*(000) = 720
*M**_r_* = 337.40	*D*_x_ = 1.229 Mg m^−^^3^
Orthorhombic, *P*2_1_2_1_2_1_	Mo *K*α radiation, λ = 0.71073 Å
Hall symbol: P 2ac 2ab	Cell parameters from 1151 reflections
*a* = 8.6000 (9) Å	θ = 2.8–18.6°
*b* = 10.7458 (11) Å	µ = 0.08 mm^−^^1^
*c* = 19.729 (2) Å	*T* = 273 K
*V* = 1823.2 (3) Å^3^	Plate, yellow
*Z* = 4	0.54 × 0.14 × 0.04 mm

### Data collection

**Table:**

Bruker SMART APEX CCD area-detector diffractometer	1955 independent reflections
Radiation source: fine-focus sealed tube	1385 reflections with *I* > 2σ(*I*)
Graphite monochromator	*R*_int_ = 0.055
ω scan	θ_max_ = 25.5°, θ_min_ = 2.1°
Absorption correction: multi-scan (*SADABS*; Bruker, 2000)	*h* = −10→10
*T*_min_ = 0.957, *T*_max_ = 0.997	*k* = −12→13
10874 measured reflections	*l* = −23→23

### Refinement

**Table:**

Refinement on *F*^2^	Primary atom site location: structure-invariant direct methods
Least-squares matrix: full	Secondary atom site location: difference Fourier map
*R*[*F*^2^ > 2σ(*F*^2^)] = 0.041	Hydrogen site location: inferred from neighbouring sites
*wR*(*F*^2^) = 0.101	H-atom parameters constrained
*S* = 1.04	*w* = 1/[σ^2^(*F*_o_^2^) + (0.0419*P*)^2^ + 0.1849*P*] where *P* = (*F*_o_^2^ + 2*F*_c_^2^)/3
1955 reflections	(Δ/σ)_max_ < 0.001
228 parameters	Δρ_max_ = 0.14 e Å^−^^3^
0 restraints	Δρ_min_ = −0.13 e Å^−^^3^

### Special details

**Table:**

Geometry. All e.s.d.'s (except the e.s.d. in the dihedral angle between two l.s. planes) are estimated using the full covariance matrix. The cell e.s.d.'s are taken into account individually in the estimation of e.s.d.'s in distances, angles and torsion angles; correlations between e.s.d.'s in cell parameters are only used when they are defined by crystal symmetry. An approximate (isotropic) treatment of cell e.s.d.'s is used for estimating e.s.d.'s involving l.s. planes.
Refinement. Refinement of *F*^2^ against ALL reflections. The weighted *R*-factor *wR* and goodness of fit *S* are based on *F*^2^, conventional *R*-factors *R* are based on *F*, with *F* set to zero for negative *F*^2^. The threshold expression of *F*^2^ > σ(*F*^2^) is used only for calculating *R*-factors(gt) *etc*. and is not relevant to the choice of reflections for refinement. *R*-factors based on *F*^2^ are statistically about twice as large as those based on *F*, and *R*- factors based on ALL data will be even larger.

### Fractional atomic coordinates and isotropic or equivalent isotropic displacement parameters (Å^2^)

**Table:**

	*x*	*y*	*z*	*U*_iso_*/*U*_eq_	
O1	0.2992 (3)	0.14972 (19)	0.03121 (11)	0.0530 (6)	
O2	0.2744 (3)	0.3535 (2)	0.01341 (12)	0.0647 (7)	
O3	−0.1643 (3)	−0.2894 (3)	0.23668 (15)	0.0871 (9)	
H3A	−0.1272	−0.2265	0.2196	0.131*	
N1	0.0397 (3)	−0.2580 (2)	0.13093 (14)	0.0542 (7)	
C1	0.3452 (4)	0.2701 (3)	0.03985 (17)	0.0491 (8)	
C2	0.4849 (4)	0.2769 (3)	0.08582 (16)	0.0501 (8)	
H2A	0.5785	0.2805	0.0576	0.060*	
C3	0.4799 (4)	0.1507 (3)	0.12056 (15)	0.0450 (8)	
H3B	0.4037	0.1550	0.1574	0.054*	
C4	0.6257 (4)	0.0914 (3)	0.14781 (18)	0.0553 (9)	
H4A	0.6661	0.1403	0.1852	0.066*	
H4B	0.7043	0.0881	0.1126	0.066*	
C5	0.5871 (4)	−0.0399 (3)	0.17203 (17)	0.0569 (9)	
H5A	0.5236	−0.0338	0.2125	0.068*	
H5B	0.6832	−0.0812	0.1845	0.068*	
C6	0.4998 (4)	−0.1229 (3)	0.11905 (16)	0.0501 (8)	
C7	0.4574 (4)	−0.2407 (3)	0.15386 (18)	0.0585 (10)	
H7A	0.5362	−0.2855	0.1750	0.070*	
C8	0.3150 (4)	−0.2855 (3)	0.15666 (17)	0.0546 (9)	
H8A	0.2984	−0.3620	0.1775	0.066*	
C9	0.1825 (4)	−0.2191 (3)	0.12821 (17)	0.0476 (8)	
C10	0.2116 (4)	−0.0959 (3)	0.09669 (17)	0.0500 (8)	
C11	0.3588 (4)	−0.0537 (3)	0.09191 (15)	0.0417 (8)	
C12	0.4121 (4)	0.0698 (3)	0.06425 (16)	0.0446 (8)	
H12A	0.4955	0.0533	0.0316	0.053*	
C13	0.4820 (5)	0.3915 (3)	0.1308 (2)	0.0783 (12)	
H13A	0.4735	0.4647	0.1031	0.118*	
H13B	0.5763	0.3953	0.1568	0.118*	
H13C	0.3945	0.3870	0.1609	0.118*	
C14	0.0693 (4)	−0.0278 (3)	0.0730 (2)	0.0805 (14)	
H14A	0.0889	0.0084	0.0294	0.121*	
H14B	0.0440	0.0367	0.1048	0.121*	
H14C	−0.0161	−0.0850	0.0697	0.121*	
C15	0.6148 (4)	−0.1591 (3)	0.0611 (2)	0.0707 (11)	
H15A	0.5614	−0.2090	0.0281	0.106*	
H15B	0.7000	−0.2056	0.0797	0.106*	
H15C	0.6536	−0.0850	0.0399	0.106*	
C16	0.0030 (4)	−0.3792 (3)	0.15327 (17)	0.0525 (8)	
C17	0.0571 (4)	−0.4863 (3)	0.12167 (19)	0.0626 (10)	
H17A	0.1292	−0.4800	0.0867	0.075*	
C18	0.0048 (5)	−0.6021 (3)	0.1418 (2)	0.0709 (11)	
H18A	0.0405	−0.6733	0.1200	0.085*	
C19	−0.0998 (5)	−0.6117 (4)	0.1940 (2)	0.0745 (12)	
H19A	−0.1337	−0.6898	0.2080	0.089*	
C20	−0.1549 (4)	−0.5067 (4)	0.2258 (2)	0.0701 (11)	
H20A	−0.2257	−0.5136	0.2612	0.084*	
C21	−0.1051 (4)	−0.3920 (3)	0.20502 (18)	0.0567 (9)	

### Atomic displacement parameters (Å^2^)

**Table:**

	*U*^11^	*U*^22^	*U*^33^	*U*^12^	*U*^13^	*U*^23^
O1	0.0561 (14)	0.0463 (13)	0.0566 (14)	−0.0012 (12)	−0.0146 (12)	0.0084 (10)
O2	0.0708 (16)	0.0514 (14)	0.0718 (16)	0.0061 (14)	−0.0142 (14)	0.0115 (12)
O3	0.0668 (18)	0.081 (2)	0.114 (2)	−0.0007 (15)	0.0180 (17)	−0.0079 (17)
N1	0.0475 (17)	0.0515 (17)	0.0638 (19)	−0.0068 (15)	0.0000 (15)	0.0104 (14)
C1	0.050 (2)	0.048 (2)	0.049 (2)	−0.0008 (18)	0.0019 (16)	0.0019 (16)
C2	0.046 (2)	0.0471 (18)	0.057 (2)	−0.0002 (17)	0.0028 (17)	−0.0032 (16)
C3	0.0446 (18)	0.0487 (18)	0.0416 (18)	−0.0018 (16)	−0.0005 (16)	0.0006 (15)
C4	0.053 (2)	0.057 (2)	0.057 (2)	−0.0018 (18)	−0.0101 (18)	0.0053 (17)
C5	0.044 (2)	0.066 (2)	0.061 (2)	−0.0025 (18)	−0.0112 (17)	0.0118 (17)
C6	0.047 (2)	0.0504 (19)	0.053 (2)	0.0012 (17)	−0.0003 (18)	0.0115 (15)
C7	0.054 (2)	0.051 (2)	0.071 (2)	0.0098 (19)	−0.0099 (19)	0.0143 (18)
C8	0.059 (2)	0.0432 (19)	0.061 (2)	0.0028 (18)	−0.0053 (19)	0.0095 (17)
C9	0.049 (2)	0.0442 (18)	0.050 (2)	−0.0004 (17)	−0.0018 (16)	0.0050 (15)
C10	0.048 (2)	0.0449 (19)	0.057 (2)	−0.0007 (17)	−0.0079 (17)	0.0057 (15)
C11	0.046 (2)	0.0399 (18)	0.0392 (18)	−0.0007 (15)	−0.0039 (15)	0.0024 (14)
C12	0.0452 (18)	0.0470 (19)	0.0415 (19)	0.0053 (16)	−0.0012 (15)	0.0054 (14)
C13	0.082 (3)	0.061 (2)	0.093 (3)	0.006 (2)	−0.027 (3)	−0.021 (2)
C14	0.052 (2)	0.060 (2)	0.129 (4)	−0.003 (2)	−0.016 (2)	0.033 (2)
C15	0.061 (2)	0.066 (2)	0.085 (3)	0.015 (2)	0.009 (2)	0.004 (2)
C16	0.0445 (19)	0.053 (2)	0.060 (2)	−0.0052 (17)	−0.0041 (18)	0.0100 (17)
C17	0.066 (2)	0.058 (2)	0.064 (2)	−0.006 (2)	0.0036 (19)	0.0054 (19)
C18	0.073 (3)	0.057 (2)	0.083 (3)	−0.011 (2)	−0.002 (3)	0.001 (2)
C19	0.064 (3)	0.064 (3)	0.095 (3)	−0.017 (2)	−0.004 (2)	0.022 (2)
C20	0.051 (2)	0.083 (3)	0.076 (3)	−0.012 (2)	0.007 (2)	0.019 (2)
C21	0.0457 (19)	0.059 (2)	0.065 (2)	−0.0004 (19)	0.0006 (19)	0.0023 (19)

### Geometric parameters (Å, º)

**Table:**

O1—C1	1.363 (4)	C8—H8A	0.9300
O1—C12	1.451 (3)	C9—C10	1.484 (4)
O2—C1	1.203 (4)	C10—C11	1.348 (4)
O3—C21	1.366 (4)	C10—C14	1.500 (5)
O3—H3A	0.8200	C11—C12	1.506 (4)
N1—C9	1.299 (4)	C12—H12A	0.9800
N1—C16	1.411 (4)	C13—H13A	0.9600
C1—C2	1.507 (5)	C13—H13B	0.9600
C2—C13	1.517 (4)	C13—H13C	0.9600
C2—C3	1.521 (4)	C14—H14A	0.9600
C2—H2A	0.9800	C14—H14B	0.9600
C3—C4	1.506 (4)	C14—H14C	0.9600
C3—C12	1.526 (4)	C15—H15A	0.9600
C3—H3B	0.9800	C15—H15B	0.9600
C4—C5	1.527 (4)	C15—H15C	0.9600
C4—H4A	0.9700	C16—C21	1.388 (5)
C4—H4B	0.9700	C16—C17	1.389 (5)
C5—C6	1.566 (4)	C17—C18	1.382 (5)
C5—H5A	0.9700	C17—H17A	0.9300
C5—H5B	0.9700	C18—C19	1.372 (5)
C6—C7	1.486 (4)	C18—H18A	0.9300
C6—C11	1.520 (4)	C19—C20	1.375 (5)
C6—C15	1.561 (5)	C19—H19A	0.9300
C7—C8	1.317 (4)	C20—C21	1.368 (5)
C7—H7A	0.9300	C20—H20A	0.9300
C8—C9	1.457 (5)		
			
C1—O1—C12	108.1 (2)	C9—C10—C14	115.3 (3)
C21—O3—H3A	109.5	C10—C11—C12	127.4 (3)
C9—N1—C16	121.4 (3)	C10—C11—C6	124.1 (3)
O2—C1—O1	120.4 (3)	C12—C11—C6	108.4 (3)
O2—C1—C2	128.9 (3)	O1—C12—C11	118.7 (3)
O1—C1—C2	110.7 (3)	O1—C12—C3	104.2 (2)
C1—C2—C13	112.3 (3)	C11—C12—C3	110.7 (2)
C1—C2—C3	101.8 (3)	O1—C12—H12A	107.6
C13—C2—C3	117.4 (3)	C11—C12—H12A	107.6
C1—C2—H2A	108.3	C3—C12—H12A	107.6
C13—C2—H2A	108.3	C2—C13—H13A	109.5
C3—C2—H2A	108.3	C2—C13—H13B	109.5
C4—C3—C2	121.0 (3)	H13A—C13—H13B	109.5
C4—C3—C12	109.7 (3)	C2—C13—H13C	109.5
C2—C3—C12	101.0 (2)	H13A—C13—H13C	109.5
C4—C3—H3B	108.2	H13B—C13—H13C	109.5
C2—C3—H3B	108.2	C10—C14—H14A	109.5
C12—C3—H3B	108.2	C10—C14—H14B	109.5
C3—C4—C5	108.8 (3)	H14A—C14—H14B	109.5
C3—C4—H4A	109.9	C10—C14—H14C	109.5
C5—C4—H4A	109.9	H14A—C14—H14C	109.5
C3—C4—H4B	109.9	H14B—C14—H14C	109.5
C5—C4—H4B	109.9	C6—C15—H15A	109.5
H4A—C4—H4B	108.3	C6—C15—H15B	109.5
C4—C5—C6	115.0 (3)	H15A—C15—H15B	109.5
C4—C5—H5A	108.5	C6—C15—H15C	109.5
C6—C5—H5A	108.5	H15A—C15—H15C	109.5
C4—C5—H5B	108.5	H15B—C15—H15C	109.5
C6—C5—H5B	108.5	C21—C16—C17	118.2 (3)
H5A—C5—H5B	107.5	C21—C16—N1	118.1 (3)
C7—C6—C11	112.6 (3)	C17—C16—N1	123.4 (3)
C7—C6—C15	106.4 (3)	C18—C17—C16	120.5 (4)
C11—C6—C15	111.7 (3)	C18—C17—H17A	119.7
C7—C6—C5	107.1 (3)	C16—C17—H17A	119.7
C11—C6—C5	109.8 (3)	C19—C18—C17	119.8 (4)
C15—C6—C5	109.1 (3)	C19—C18—H18A	120.1
C8—C7—C6	124.0 (3)	C17—C18—H18A	120.1
C8—C7—H7A	118.0	C18—C19—C20	120.4 (4)
C6—C7—H7A	118.0	C18—C19—H19A	119.8
C7—C8—C9	122.1 (3)	C20—C19—H19A	119.8
C7—C8—H8A	118.9	C21—C20—C19	119.6 (3)
C9—C8—H8A	118.9	C21—C20—H20A	120.2
N1—C9—C8	124.5 (3)	C19—C20—H20A	120.2
N1—C9—C10	117.6 (3)	O3—C21—C20	118.3 (3)
C8—C9—C10	117.8 (3)	O3—C21—C16	120.4 (3)
C11—C10—C9	119.2 (3)	C20—C21—C16	121.3 (3)
C11—C10—C14	125.5 (3)		
			
C12—O1—C1—O2	175.7 (3)	C14—C10—C11—C6	177.4 (3)
C12—O1—C1—C2	−5.8 (3)	C7—C6—C11—C10	−2.1 (5)
O2—C1—C2—C13	34.0 (5)	C15—C6—C11—C10	117.5 (4)
O1—C1—C2—C13	−144.4 (3)	C5—C6—C11—C10	−121.3 (3)
O2—C1—C2—C3	160.4 (3)	C7—C6—C11—C12	174.2 (3)
O1—C1—C2—C3	−18.0 (3)	C15—C6—C11—C12	−66.2 (3)
C1—C2—C3—C4	153.7 (3)	C5—C6—C11—C12	54.9 (3)
C13—C2—C3—C4	−83.3 (4)	C1—O1—C12—C11	151.1 (3)
C1—C2—C3—C12	32.6 (3)	C1—O1—C12—C3	27.3 (3)
C13—C2—C3—C12	155.6 (3)	C10—C11—C12—O1	−8.0 (5)
C2—C3—C4—C5	−173.5 (3)	C6—C11—C12—O1	175.9 (3)
C12—C3—C4—C5	−56.6 (3)	C10—C11—C12—C3	112.5 (4)
C3—C4—C5—C6	51.6 (4)	C6—C11—C12—C3	−63.6 (3)
C4—C5—C6—C7	−173.9 (3)	C4—C3—C12—O1	−166.0 (2)
C4—C5—C6—C11	−51.4 (4)	C2—C3—C12—O1	−37.2 (3)
C4—C5—C6—C15	71.3 (4)	C4—C3—C12—C11	65.3 (3)
C11—C6—C7—C8	4.8 (5)	C2—C3—C12—C11	−165.9 (3)
C15—C6—C7—C8	−117.8 (4)	C9—N1—C16—C21	126.8 (4)
C5—C6—C7—C8	125.6 (4)	C9—N1—C16—C17	−60.1 (5)
C6—C7—C8—C9	−3.2 (6)	C21—C16—C17—C18	−0.4 (5)
C16—N1—C9—C8	−9.8 (5)	N1—C16—C17—C18	−173.5 (3)
C16—N1—C9—C10	173.1 (3)	C16—C17—C18—C19	−0.9 (6)
C7—C8—C9—N1	−178.5 (4)	C17—C18—C19—C20	1.0 (6)
C7—C8—C9—C10	−1.4 (5)	C18—C19—C20—C21	0.1 (6)
N1—C9—C10—C11	−178.8 (3)	C19—C20—C21—O3	178.7 (4)
C8—C9—C10—C11	3.9 (5)	C19—C20—C21—C16	−1.4 (6)
N1—C9—C10—C14	1.7 (5)	C17—C16—C21—O3	−178.6 (3)
C8—C9—C10—C14	−175.6 (3)	N1—C16—C21—O3	−5.1 (5)
C9—C10—C11—C12	−177.6 (3)	C17—C16—C21—C20	1.5 (5)
C14—C10—C11—C12	1.9 (6)	N1—C16—C21—C20	175.0 (3)
C9—C10—C11—C6	−2.0 (5)		

### Hydrogen-bond geometry (Å, º)

**Table:**

*D*—H···*A*	*D*—H	H···*A*	*D*···*A*	*D*—H···*A*
O3—H3*A*···N1	0.82	2.28	2.747 (4)	116
